# The effect of trichostatin-A and tumor necrosis factor on expression of splice variants of the MaxiK and L-type channels in human myometrium

**DOI:** 10.3389/fphys.2014.00261

**Published:** 2014-07-15

**Authors:** Sarah L. Waite, Saurabh V. Gandhi, Raheela N. Khan, Neil R. Chapman

**Affiliations:** ^1^Academic Unit of Reproductive and Developmental Medicine, Department of Human Metabolism, University of SheffieldSheffield, UK; ^2^Department of Obstetrics and Gynaecology, Sheffield Teaching Hospitals NHS Foundation TrustSheffield, UK; ^3^Division of Medical Sciences and Graduate Entry Medicine, School of Medicine, Royal Derby Hospital, University of NottinghamDerby, UK

**Keywords:** parturition, MaxiK, LTCC, splice variants, TNF, TSA

## Abstract

The onset of human parturition is associated with up-regulation of pro-inflammatory cytokines including tumor necrosis factor (TNF) as well as changes in ion flux, principally Ca^2+^ and K^+^, across the myometrial myocytes membrane. Elevation of intra-cellular Ca^2+^ from the sarcoplasmic reticulum opens L-type Ca^2+^ channels (LTCCs); in turn this increased calcium level activates MaxiK channels leading to relaxation. While the nature of how this cross-talk is governed remains unclear, our previous work demonstrated that the pro-inflammatory cytokine, TNF, and the histone deacetylase inhibitor, Trichostatin-A (TSA), exerted opposing effects on the expression of the pro-quiescent Gαs gene in human myometrial cells. Consequently, in this study we demonstrate that the different channel splice variants for both MaxiK and LTCC are expressed in primary myometrial myocytes. MaxiK mRNA expression was sensitive to TSA stimulation, this causing repression of the M1, M3, and M4 splice variants. A small but not statistically significantly increase in MaxiK expression was also seen in response to TNF. In contrast to this, expression of LTCC splice variants was seen to be influenced by both TNF and TSA. TNF induced overall increase in total LTCC expression while TSA stimulated a dual effect: causing induction of LTCC exon 8 expression but repressing expression of other LTCC splice variants including that encoding exons 30, 31, 33, and 34, exons 30–34 and exons 40–43. The significance of these observations is discussed herein.

## Introduction

In the developed world, premature birth (that before 37 weeks completed gestation) complicates 6–12% of pregnancies (Khashan et al., 2010). Annually it is estimated that 1.1 million babies worldwide die from being born prematurely (Blencowe et al., 1990; Chang et al., 2013), surviving infants having an elevated risk of major long-term mental and physical handicap (Marlow et al., 2005; Costeloe et al., 2012). Moreover, such infants also have a disproportionate effect on health-care budgets world-wide: a recent UK estimate of the total cost of preterm birth to the public sector was £2.95 billion (Mangham et al., 2009). Tocolytic therapies (drugs which stop premature contractions of the womb) are few in number and are associated with complications for both infant and mother (Oei, 2006). Since the antenatal health of a baby is a major predictor of adult morbidity (Nijland et al., 2008), reducing the incidence of premature birth is an unequivocal healthcare goal. Indeed, a recent review suggested a conservative target of relative reduction of the pre-term birth rate by 5% by 2015 (Chang et al., 2013). Until the fundamental biology of the pregnant human uterus is defined, however, that target is unlikely to be realized.

The smooth muscle of the non-pregnant uterus, the myometrium, naturally contracts when subjected to stretch. During normal gestation, however, the pregnant myometrium must stretch to accommodate the growing baby. As such, the myometrium enters a prolonged phase of relaxation which is termed quiescence. This quiescent state is important because it facilitates normal growth and development of the baby. The quiescent state is characterized by slow wave potentials where the membrane potential cycles between depolarization and repolarizations without reaching the threshold level to produce an action potential (Parkington et al., 1999; Smith et al., 2002; Aguilar and Mitchell, 2010). At term, this quiescent state ends and these slow wave potentials become frequent synchronized action potentials during which the membrane potential rapidly rises and falls (Wilde and Marshall, 1988)—i.e., the myometrium undergoes the rhythmic contractions seen in labor to ensure the baby is delivered (Khan et al., 1993b, 1997, 1998a,b; Tribe et al., 2000; Shmygol et al., 2007). Unfortunately, the fundamental nature of the molecular mechanisms regulating human quiescence and labor are poorly understood, the corollary being that this greatly limits our understanding of how parturition begins too early leading to premature birth of the baby.

In terms of myometrial quiescence, this period of pro-longed relaxation occurs when calcium is maintained below the threshold for depolarization that would trigger calcium entry. One mechanism by which this is achieved involves potassium channels that either repolarize the cell membrane thus causing the calcium channels to close and therefore prevent further calcium from entering the cell (Khan et al., 1993a,b, 1997; Shmygol et al., 2007) or maintaining the cell resting membrane potential through potassium leak channels of the two-pore domain potassium (K2P) channel family (Buxton et al., 2010) and others (Knock and Aaronson, 1999; Tribe et al., 2000). Calcium activated potassium channels, including MaxiK, have an important role regulating action potentials in some smooth muscles. Calcium influx through L-type Ca^2+^ channels (LTCCs) is the principle source driving smooth muscle contractility and while many types of potassium channels counteract the influx of calcium and maintain or promote smooth muscle relaxation, the large-conductance calcium-activated potassium channel (BKCa, MaxiK) is activated by intracellular calcium and acts to oppose the depolarization (Toro et al., 1990; Khan et al., 1993b, 1997) and so responds to both changes in calcium concentration and membrane potential. Calcium influx through LTCCs is the principle source driving smooth muscle contractility and therefore calcium activated potassium channels, including MaxiK, have an important role regulating action potentials in some smooth muscles. Thus, fine tuning of the activity of both MaxiK and LTCC would appear to be, in part, a key process for maintenance of quiescence during gestation and contraction during labor (Khan et al., 1993b, 1997, 1998a,b; Brainard et al., 2005; Shmygol et al., 2007).

The MaxiK is thought to be one of the key potassium channels within the myometrium (Anwer et al., 1993; Khan et al., 1993b, 1997, 1998a,b; Perez et al., 1993; Tribe et al., 2000; Shmygol et al., 2007). The MaxiK channel itself is a tetrameric structure containing a pore-forming α-subunit and a regulatory β-subunit (Wallner et al., 1995; Lu et al., 2006) and is documented to have a number of splice variants (Song et al., 1999; Curley et al., 2004). Briefly, these include the MK44 variant (M1) which can either encode a 132 bp insert between exons 1 and 2 or have this insert omitted. The channels containing the insert have diminished sensitivity to intracellular calcium and also voltage (Korovkina et al., 2001). The second variant involves the mutually exclusive use of either exons 10 and 11 or 11 and 12 (M2), which can cause a truncation of the channel protein (Curley et al., 2004). Three variants of exon 19 have also been described these are a 3′ truncation of the exon, skipping of the exon and the use of the entire of exon 19, (M3), the effect of these different variants has not been fully explored (Curley et al., 2004). The Stress Regulated Exon (STREX/M4) is another well described variant which involves the differential utilization of exons 22 and 23, channels utilizing both exons have been shown to have increased mechano-sensitivity and hypoxia inhibition (McCartney et al., 2005; Lu et al., 2006; Wang et al., 2010). Finally there is a variant involving the use or omission of exon 29, (M5), utilization of exon 29 creates channels with increased activation rates and modified calcium co-operativity (Ha et al., 2000; Yan et al., 2008).

It is clear that alternate splicing of the MaxiK α-subunit can alter its sensitivity to calcium, voltage, protein phosphorylation and cellular localization: all are methods by which the diversity of K^+^ channel signaling arises (Soloff et al., 2004; Torres et al., 2007; Aguilar and Mitchell, 2010). This provides a mechanism for fine tuning the channels response to a diverse range of regulatory stimuli. Indeed the expression of different MaxiK channel isoforms is tissue and stimulus-specific (Lu et al., 2006) and may be a mechanism by which uterine contractility can be modulated during gestation.

The LTCC is the predominant calcium channel in the myometrium (Tezuka et al., 1995; Parkington et al., 1999; Collins et al., 2000). The LTCC is a type of voltage-dependent calcium channel consisting of five subunits (α1, α2, β, γ, and δ) with the α1-subunit defining most of the channel's properties (Jurkat-Rott and Lehmann-Horn, 2004; Aguilar and Mitchell, 2010). LTCC are responsible for normal myocardial and vascular smooth muscle contractility and are also subject to splicing similar to MaxiK (Jurkat-Rott and Lehmann-Horn, 2004; Tang et al., 2004; Aguilar and Mitchell, 2010). Briefly, these include the mutually exclusive use of exons 1, 1b, or 1c (L1), these affect membrane expression of the channel (Snutch et al., 1991; Soldatov, 1992; Bannister et al., 2011). Exons 8 and 8^*^ are also mutually exclusively expressed (L4), use of exon 8^*^ results in channels with lower Dihydropyridine (DHP) block sensitivity, more rapid activation and slower inactivation rates (Soldatov, 1992). Exons 30, 31, 32, 33, and 34 (L10) can also be utilized in a number of different combinations (L10). These alter the size and rigidity of the linker between the S3 and S4 segments of the channel, with a shorter linker resulting in channels with slower gating kinetics and longer one resulting in channels with faster gating kinetics (Perez-Reyes et al., 1990; Yang et al., 2000). Finally, splice site 11/12 (L11/12) includes exons 40, 41, 42, and 43, variants in this region include the use of exon 40A (exon 40 with a 19 bp deletion), exon 40B (exon 40 with a 125 bp addition), exon 41A and exon 43 with an additional 132 bp (Gerhardstein et al., 2000). These variations affect calmodulin binding, calcium dependant inactivation and the presence or absence of a cAMP dependant protein kinase A site (Perez-Reyes et al., 1990; Snutch et al., 1991; Diebold et al., 1992; Soldatov, 1992, 1994; Tang et al., 2004).

Given the abundance of MaxiK in the myometrium and its links with Ca^2+^ flux via the LTCC, it is clear that these channel families play a pivotal role in governing myometrial quiescence and subsequent phenotypic change to uterine contractility. By responding to calcium influx the MaxiK channels provide an effective mechanism of negative feedback of to maintain relaxation (Khan et al., 1993b, 1997, 1998b, 2001). Moreover, we believe that the balance between LTCCs and MaxiK channels determines whether quiescence or contractility occurs within the myometrium.

In the human myometrium, the cessation of uterine quiescence and the onset of both normal and preterm labor are associated with a number of pro-inflammatory cytokines, including, but not limited to, IL-1β, tumor necrosis factor (TNF), and IL-8, which are regulated by a family of transcription factors collectively referred to as Nuclear Factor kappaB (NFκB; reviewed in Cookson and Chapman, 2010). Our pilot promoter array studies demonstrate that the genomic regulatory regions of both MaxiK and the LTCC are bound by NFκB RelA in response to TNF (Cookson, 2010). Moreover, TNF was seen to induce nuclear localization of both channels in primary myometrial cells.

We and others have previously demonstrated that external agents such as TNF (potent pro-inflammatory cytokine) can induce myometrial contractility while other compounds, namely trichostatin-A (TSA), can promote myometrial relaxation in isolated human smooth muscle strips (Lu et al., 1999; Moynihan et al., 2008; Webster et al., 2013). While the exact means by which this process occurred could not be elucidated, that study also demonstrated that TSA caused an up-regulation of the GTP-binding protein, Gαs, while TNF had the reciprocal function and repressed the Gαs promoter. The repression was due to recruitment of an enzyme, histone deacetylase-1 (HDAC-1) to the regulatory region of the Gαs gene (Webster et al., 2013). Together, these data illustrated that while TSA can induce a relaxed myometrial phenotype required to stop muscle contraction, it could not overcome the uterine inflammation induced by those pro-inflammatory mediators, including TNF, known to be present in the uterus during labor.

In our view, our previous data suggests that there is a molecular conflict between TSA and TNF which we believe involves ion channel function possibly through the Gαs subunit. Indeed, both MaxiK and LTCC are linked to adenylyl cyclase activity and β2-adrenergic receptor function (Toro et al., 1990; Carney et al., 2002; Chanrachakul et al., 2004; Liu et al., 2004; Ledoux et al., 2006; Torres et al., 2007) which, when engaged, promote smooth muscle relaxation through increased Gαs activity, subsequent elevations in intra-cellular cAMP and efflux of K^+^ via MaxiK (Chanrachakul et al., 2004; Aguilar and Mitchell, 2010). It is likely, however, that both agents would also influence both ion channel expression and subsequent RNA splicing, which, in turn, would also modulate myometrial contractility. Given the effects we have previously reported for both TNF and TSA on primary human myometrium and myometrial myocytes (Webster et al., 2013), in this study we wished to examine the expression, localization, and splicing pattern of both the MaxiK channel and the LTCC in primary human myometrial cells and define whether these parameters were influenced by TNF and TSA.

## Materials and methods

### Myometrial biopsy collection

All women were recruited at the Department of Obstetrics and Gynaecology at the Jessop Wing Hospital for Women, Sheffield. Informed consent was obtained from all patients and approved by the Leeds Bradford Local Research Ethics Committee (Ref No. 12/YH/0229). Lower segment myometrial biopsies were taken from healthy women undergoing elective cesarean sections at term (*n* = 45, age 16–43, gestation 37–40 weeks) as described previously. Myometrial smooth muscle cell cultures were then subsequently generated as detailed in Phaneuf et al. (1993).

### Immunohistochemical staining for the MaxiK and L-Type Ca^2+^ channels

A single myometrial biopsy was used to generate each individual primary culture. Myometrial cells were cultured in a 6-well plate for 2–4 passages. Control cells were human oral fibroblasts (a kind gift from Dr. Vanessa Hearnden, Human Nutrition Unit, University of Sheffield). Upon reaching 80% confluence the cells were harvested from two matched cultures from the same biopsy. One of these underwent immunohistochemistry and the second, RNA extraction. For immunohistochemistry, cells were fixed in 4% (w/v) paraformaldehyde overnight at 4°C. Prior to staining the cells were washed 3 times for 5 min in PBS and permeabilized with PBS containing 1% (w/v) BSA endogenous peroxidase was quenched with 3% (v/v) hydrogen peroxide for 10 min. Staining was then carried out using the Vectastain® *Elite* ABC Kit according to the manufacturer's instructions. Cells were stored in PBS and images capture using a Nikon DS-Fil Microscope camer and Olympus CKX41 microscope.

### Collagen gel contraction assay

Human myometrial cells were included in collagen gels as detailed in Ngo et al. (2006) Briefly, confluent cells were harvested and resuspended in DMEM with D-valine. A type-I collagen solution (3 mg/ml in 0.1% acetic acid) was adjusted to pH 7.2 with 1 M NaOH. The final concentration of collagen was 1.5 mg/ml to which myometrial cells were then added. The collagen gel:cell suspension was incubated in a 24 well plate for 20 min to allow gelling, then 0.5 ml DMEM with D-valine medium was added over the collagen lattice.

Upon reaching 80% confluence the collagen gel was dissociated from the sides and bottom of the well. Then the plate was gently swirled to ensure the gel was free floating. Myometrial cells were then stimulated with 10 ng/ml TNF or 2 μg/ml TSA, or left un-stimulated. PHM1-31 cells were used as contraction-positive controls while HEK293 cells were used as contraction negative controls (Chevillard et al., 2007; Fitzgibbon et al., 2009). At pre-determined time points the plate was removed from the incubator for image capture and acquisition (SynGene GBox Chemi-16 gel documentation system). Subsequent surface area quantification was undertaken using GeneTools Version 4 quantification software (SynGene, Cambridge UK). Briefly, this software was used to trace the outline of the gel at each time point and the mean pixel area calculated. The surface area at each time point is reported as a percentage of the initial gel surface area in that well, a decrease in surface area indicates contraction whilst an increase indicates relaxation.

### RNA extraction and cDNA synthesis

On reaching 90% confluence the cells were stimulated with 10 ng/ml TNF for 1 h or 2 μg/ml TSA for 24 h with non-stimulated cells serving as a control. RNA was extracted from the cells using the EZRNA extraction kit (Geneflow Ltd. Staffordshire, UK) according to the manufacturer's instructions. The extracted RNA was quantified using the nanophotometer (Implen, Germany). cDNA synthesis was performed using iScript cDNA synthesis Kit (Bio-Rad; Hertfordshire UK) according to the manufacturer's guidelines. One microlitre of cDNA was used per PCR.

### Polymerase chain reaction

PCR was carried out using PCR Master Mix (Promega; Southampton UK) according to the manufacturer's guidelines. For characterization of the primary cultures Actin α2 (NM_001141945.1) was chosen as an indicator of the presence of myocytes and Thy-1 cell surface antigen (NM_006288.3), as an indicator of the presence of fibroblasts. Primers were designed to amplify each of the RNAs (Table 1) PHM1 RNA was used as a positive control for actin α2 and a negative control for Thy-1, Fibroblast RNA was used as a positive control for Thy-1.

**Table 1:** **Sequences of primers employed in RT-PCR experiments in this study**.

**Channel/region**	**Primer name and sequence**		**Size (bp)**	
actin α2	Forward	5′-tggcttggcttgtcagggcttg-3′	239	
	Reverse	5′-cgggtgctcagaacgctgga-3′		
Thy-1	Forward	5′-ctgggtgcagcaaccggagg-3′	307	
	Reverse	5′-tgctcaggcacccccacagt-3′		
GAPDH	Forward	5′-ctgccgtctagaaaaacc-3′	214	
	Reverse	5′-ccaccttcgttgtcatacc-3′		
MaxiK	Forward	5′- cggaggcagcagtcttag-3′	242	
	Reverse	5′- aagaaagtcaccatggaggag-3′		
MaxiK	Forward	5′-ctcctccatggtgactttctt-3′	437/305	
132 (M1)	Reverse	5′-ttacaagtgcaccgatgctg-3′		
MaxiK	Forward	5′-ggaaaccgcaagaaatac-3′	565	
2 (M2)	Reverse	5′-acctcatggagaagaggttg-3′		
MaxiK	Forward	5′-ggtctgtccttccctactgt-3′	547	
srkr (M3)	Reverse	5′- caaagatgcagaccacgaca-3′		
MaxiK	Forward	5′- gtgccagcaactttcattac-3′	622/535	
strex (M4)	Reverse	5′- tcagggtcatcatcatcgtc-3′		
MaxiK 5 (M5)	Forward	5′- acagcatttgccgtcagtg-3′	857	
	Reverse	5′- aatattcaaggcagacaaag-3′		
L-Type	Forward	5′- gccctatgtggccctcctgatcgtgat-3′	956	
	Reverse	5′- cttgtccagctcctcctcagcggtgaga-3′		
L 1b/1c	Forward	5′-ggatgtacattccagaggaaa-3′ b	329b/324c	
	Reverse	5′- ctctgctgtgctctggactgt-3′ c		
	Reverse	5′- ggccctccggatggggttct-3′ b/c		
L 8/8* (L4)	Forward	5′- cagtgccagaacggcacggt-3′	244	
	Reverse	5′- cgctcaacacaccgagaacca-3′ 8		
	Reverse	5′- cgctaagcacaccgagaacca-3′ 8*		
L 31 (L10)	Forward	5′- ggaatacgccctcaaggcccg-3′	30/32/33/34	343
	Reverse	5′- gggagagcattgggtatgttcagc-3′	30/32/34	259
			30/31/33/34	370
			30/31/32/33/34	454
L 41	Forward	5′- tggtccatccttggtccccacc-3′	40-/40b/43+	643
(L11/12)	Reverse	5′- agcagcggacacagcctcct-3′	40/41+/42/43	672
			40/41/42/43	615

For the detection of channel splice variants within the primary cultures, primers were selected to cover an un-spliced region in each channel and also to cover a number of spliced regions in each channel. The individual PCR primer sequences are detailed in Table 1; individual splice variant-specific PCR conditions are listed in Table 2. PCRs were performed using a Sensoquest Labcycler (Geneflow Ltd., Staffordshire, UK). All channel RT-PCR reactions were done simultaneously and with the GAPDH control serving experiments for both MaxiK and LTCC channels. Consequently **Figure 3D** is duplicated in **Figure 4D** purely for this reason. The PCR products were analyzed by agarose gel electrophoresis. The separated DNA fragments were visualized using UV light (wavelength 310 nm). Images were captured using the SynGene GBox Chemi-16 gel documentation system. Subsequent band quantification and gel calibration with DNA ladders (LowRanger Ladder, 2000–100 bp Geneflow Ltd. Staffordshire, UK) was undertaken using GeneTools Version 4 quantification software (SynGene, Cambridge UK).

**Table 2:** **Reaction conditions employed for all channel RT-PCR experiments in this study**.

**Channel/ region**	**Annealing temp. (°C)**	**Duration of denature/ anneal/elongation cycle (seconds)**	**Number of cycles**
MaxiK	52	20/45/60	35
MaxiK 132 (M 1)	54	25/30/45	35
MaxiK 2 (M 2)	50	25/30/45	35
MaxiK srkr (M 3)	54	25/30/45	35
MaxiK strex (M 4)	52	25/30/45	35
MaxiK 5 (M 5)	53	25/30/45	35
L-Type	63	30/60/30	40
L 8/8* (L4)	59	25/30/45	40
L 31 (L10)	59	25/30/45	40
L 41 (L11/12)	59	25/30/45	40

### Analysis of PCR products

After manual band quantification of the splice variant PCR analysis was performed as follows: first each individual sample set and PCR reaction were analyzed separately. Within each sample set and PCR reaction the un-stimulated sample was quantified as 100% the stimulated samples for the matching PCR reactions were then quantified as a percentage of the un-stimulated reaction. The exception to this, however, was quantification of the LTCC exon-8 variant after TSA treatment. Since unstimulated cultures and those exposed to TNF did not express exon-8 in our experiments, we have needed to show the data as arbitrary units as we cannot give data as a percentage increase from a zero value. All experiments were repeated between three and eight times and results are expressed as the mean ± *SD*. Each repeat represented one individual myometrial biopsy. All data analyses were conducted on GraphPad Prism Version 5.02 (GraphPad Software, San Diego, California) where a one way ANOVA for matched samples with Dunett's post-test was performed to compare the individual stimulations against the un-stimulated control; p<0.05 was considered statistically significant.

## Results

### Characterization of primary cultures

**Figure 1:**
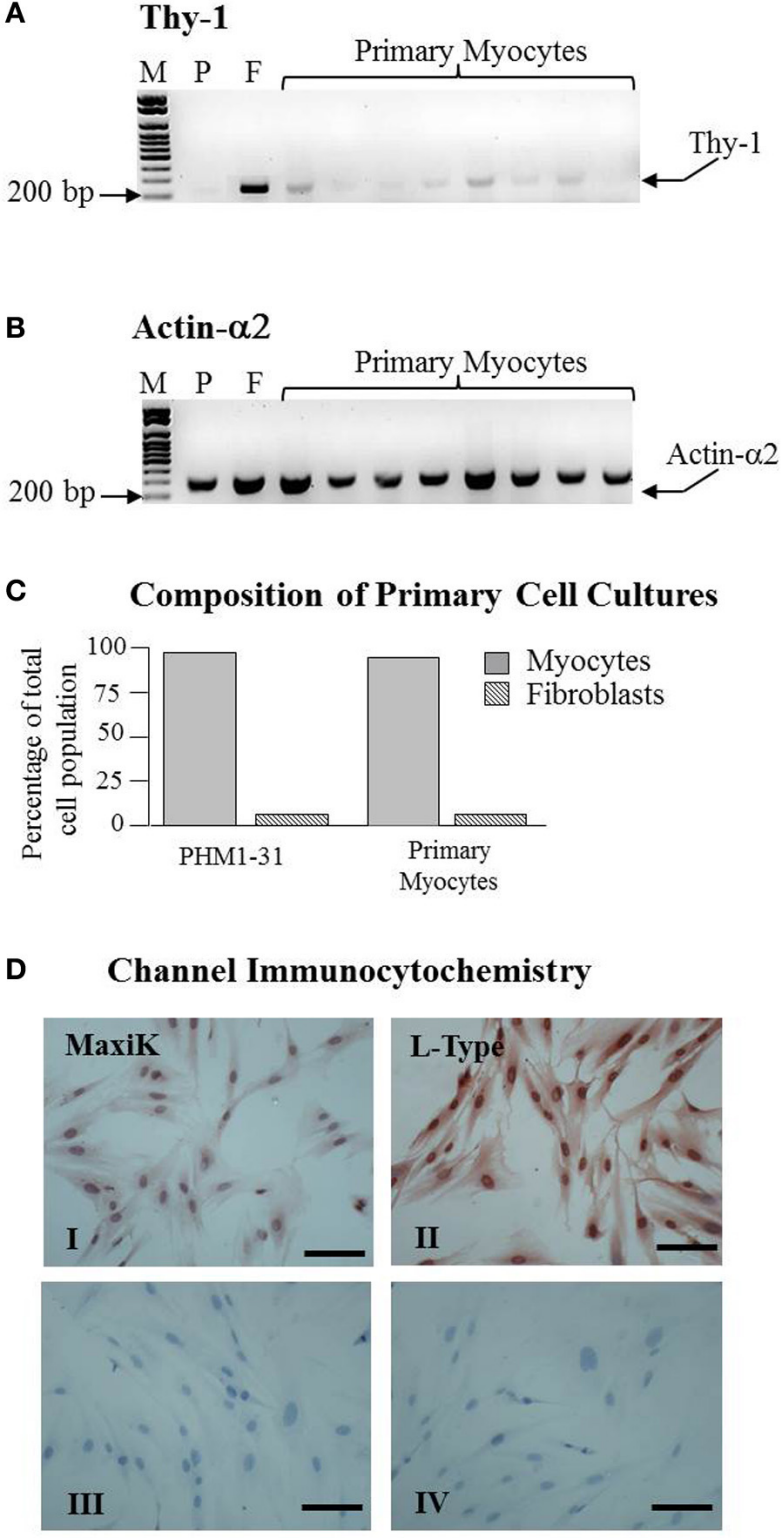
**Primary myometrial cell cultures are composed primarily of myocytes and express both the MaxiK and L-type Ca^2+^ channels.** RNA from primary myometrial cell cultures was extracted and amplified by PCR. **(A)** Primary myometrial cell cultures express a low level of Thy-1 mRNA indicating a low level of Fibroblasts in the culture. **(B)** Primary myometrial cell cultures express a high level of Actin-α2 mRNA indicating a high level of myocytes in the culture. Manual quantification of the relative band intensities was used to confirm the presence of myocytes and to estimate the level of fibroblast contamination. **(C)** Primary myometrial cell cultures are comprised of 88–98% myocytes (gray bar) and between 1.2 and 11% fibroblasts (striped bar) which is comparable to the PHM1 cultures. **(D)** Myometrial cells were staining using antibodies specific to MaxiK (I) and L-Type (II) channels. Controls excluded primary (III) and secondary antibody (IV). Dark red/brown staining denotes specific staining of the protein of interest (scale bar is 100 μm).

Our aim was to determine if TNF and TSA exerted their effect on myometrial smooth muscle contractility via the regulation of the expression of either the MaxiK and LTCCs or their splice variants. As a first step we characterized the primary cells derived from the biopsies to verify their suitability as samples for this purpose. To this end we first established that our primary cells cultures consisted primarily of myocyte cells rather than fibroblasts. This was done by measuring the expression of Actin α2, a smooth muscle actin and Thy-1 a cell surface antigen expressed specifically from fibroblasts. An immortalized human myometrial cell line PHM1-31 was used as a smooth muscle positive control and oral fibroblasts as a fibroblast cell positive control. PCR using Thy-1, showed only faint bands (Figure 1A) in the PHM1-31 cell line and the primary cultures indicting there is only a low level of fibroblast or myofibroblast contamination. In contrast to this and as expected, the fibroblast culture showed a much more intense Thy-1 band (Figure 1A). All cultures gave an intense band when Actin α2 primers were employed in the PCR indicating the presence of smooth muscle cells (Figure 1B). Importantly, within the primary myocyte cultures the level of fibroblast contamination was found to vary between 1.2 and 11% which is comparable with the immortalized PHM1-31 cell line (Figure 1C).

### Channel immunocytochemistry

Culturing of primary cells has been shown to be able to affect certain functional properties of the cells such as the loss of steroid receptors (Berns et al., 1985; Tyagi et al., 2006). The effect of culturing on ion channel expression is unclear. Consequently it was important to determine if the primary myocyte cultures expressed the MaxiK and LTCCs. Immunocytochemistry demonstrated that both channel types remain present in primary myometrial cell cultures (MaxiK—Figure 1D, Panel I; LTCC – Dig. 1D, Panel II. Antiserum controls Figure 1D, Panels III-IV). Interestingly, intense nuclear staining was observed for both channels although the significance of this remains unclear at present.

### Primary myocyte contractility

**Figure 2:**
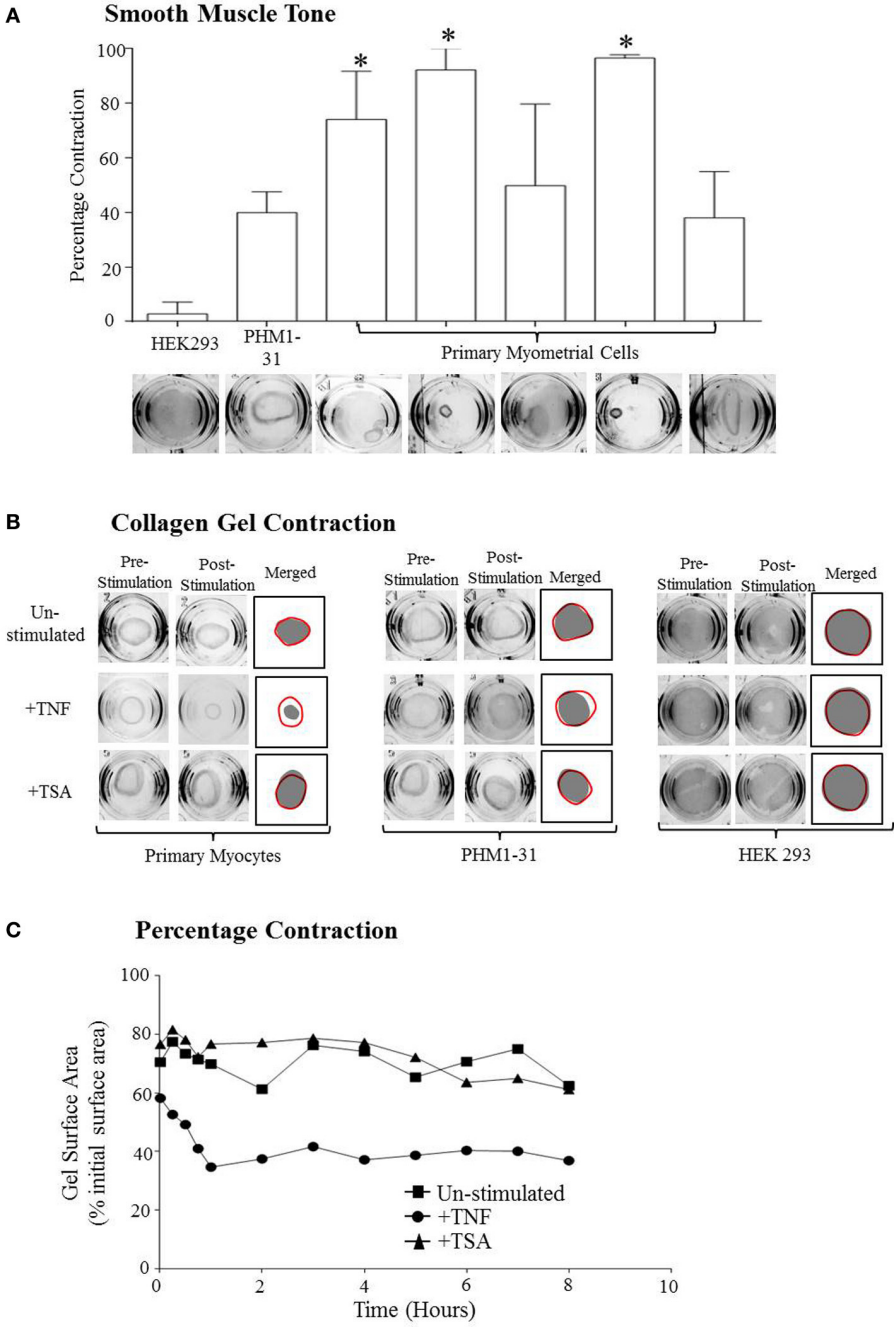
**Primary myometrial cell cultures retain smooth muscle tone and the ability to contract. (A)** The reduction of the surface area of the collagen after release demonstrates the retention of smooth muscle tone in the primary myometrial cell cultures. In the more confluent cultures this reduction in gel size becomes significant (^*^p < 0.05) There is no reduction in gel size in the HEK293 cultures. **(B)** Depiction of the maximum gel contraction for each culture after stimulation. **(C)** Depiction of the change in collagen gel surface area of the primary cells over the course of the experiment.

The final step in characterizing the primary cultures was determining if they retained the ability to contract. A collagen contraction assay was used for this purpose. Primary cells were embedded within collagen gels and either left un-stimulated or stimulated with TNF or TSA. PHMI-31 cells and HEK293 cells left un-stimulated or stimulated with TNF or TSA were used as positive and negative controls respectively. The area of the gels was recorded to determine what effects the stimulants had on the contractility of the cells. A reduction in the surface area of the gel after the gel was released was an indication that the cells retained smooth muscle tone, i.e., the resistance to passive stretch during resting state. Upon the release of the gels from the sides of the well there was a visible reduction in the size of the gels containing both primary cells and PHM1-31 cells, there was no reduction in gel size seen for the HEK293 cells. This demonstrates that the primary cells still retain contractile tone. The amount of contraction was calculated as the inverse of the gel size after release expressed as a percentage of the original gel size (Figure 2A).

In the collagen gels containing HEK293 cells, no contraction was observed upon treatment with TNF (Figure 2B). In contrast to this, however, when collagen embedded PHM1-31 or primary cells, where stimulated with TNF, there was a significant reduction in gel surface area afterwards suggesting that TNF was inducing cell contraction (Figures 2B,C). Significantly, an increase in gel surface area was observed when cultures were treated with TSA indicating that it induced a loss of basal tone in the collagen-embedded myocyte cultures although this did not reach statistical significance (Figures 2B,C).

### MaxiK splice variants are expressed in non-laboring term pregnant myometrium and are down-regulated by trichostatin-A

**Figure 3:**
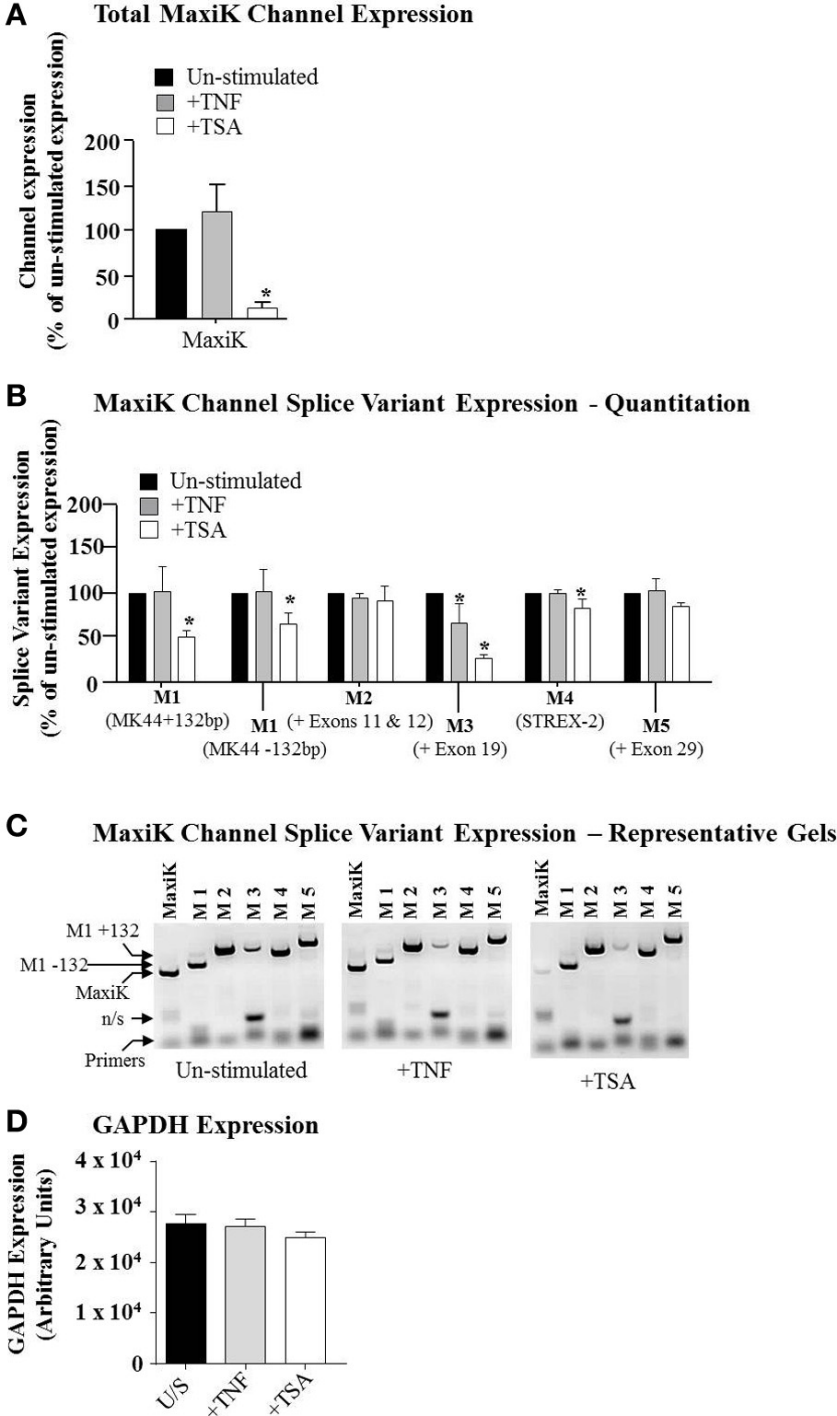
**The expression of the MaxiK channel and splice variants in primary myometrial cell cultures.** Primary myometrial cell cultures were stimulated with 10 ng/ml TNF for 1 h, 100 ng/ml TSA for 24 h or left un-stimulated. RNA was extracted, reverse transcribed and amplified using channel and splice variant specific primers. Manual quantification of the relative band intensities were used to quantify the level of expression and this was expressed as a percentage of the un-stimulated expression. **(A)** TNF had no significant effect whilst TSA significantly reduced the expression of the MaxiK channel (^*^p < 0.05). **(B)** Primary Myometrial cell cultures express a range of MaxiK splice variants, TNF had no effect on splice variant expression and TSA significantly reduced the expression of the MK44 splice variant (^*^p < 0.05). **(C)** Representative gel of the RT-PCR products. **(D)** Neither TNF nor TSA influenced the expression of GAPDH.

**Figure 4:**
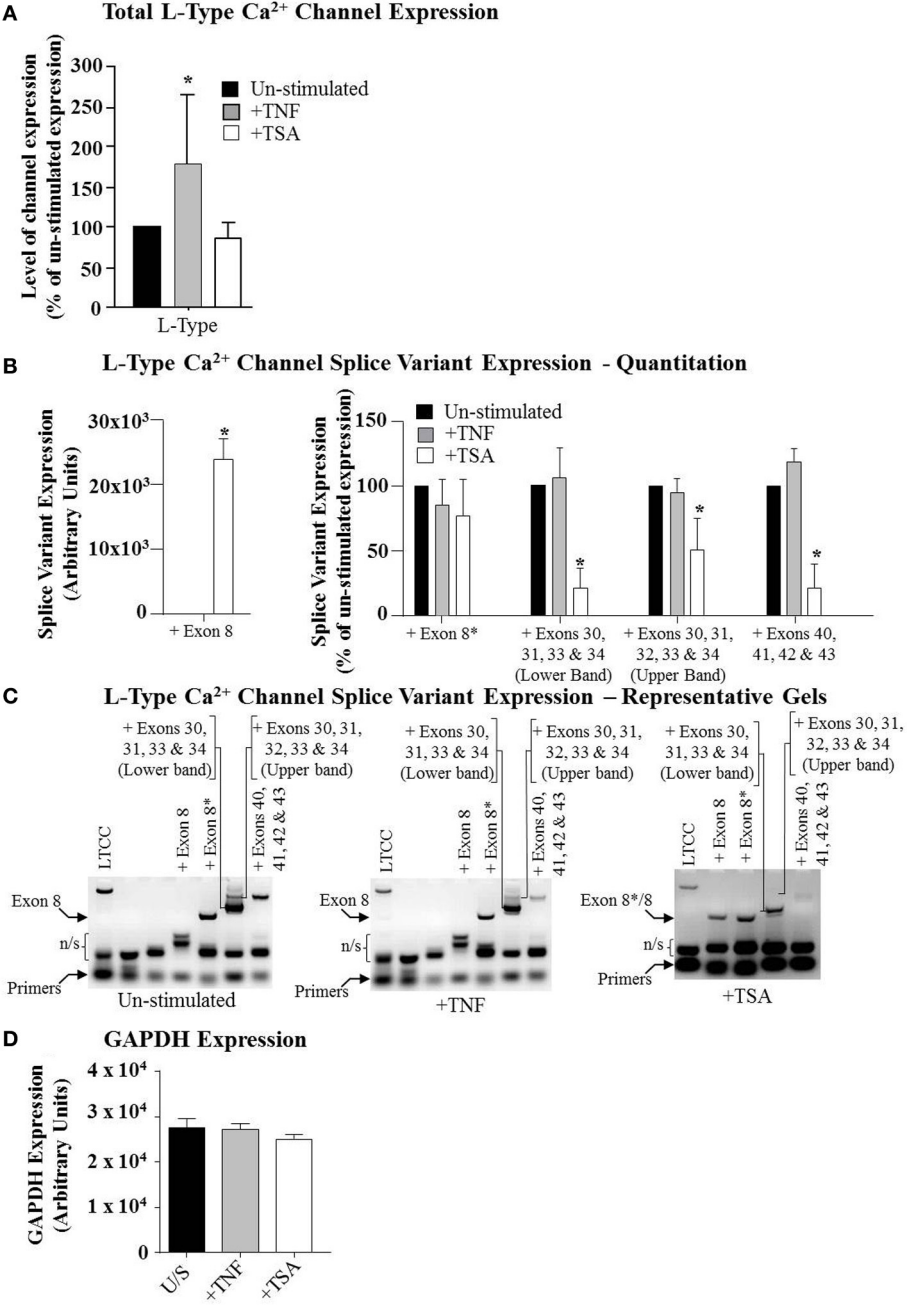
**The expression of the L-Type Ca^2+^ channel and splice variants in primary myometrial cell cultures.** Primary myometrial cell cultures were stimulated with 10 ng/ml TNF, 100 ng/ml TSA or left unstimulated. RNA was extracted, reverse transcribed and amplified using channel and splice variant specific primers. Manual quantification of the relative band intensities were used to quantify the level of expression and this was expressed as a percentage of the un-stimulated expression. **(A)** TNF significantly increased the expression of the L-Type Ca^2+^ channel whilst TSA had no significant effect (^*^p < 0.05). **(B)** Primary myometrial cell cultures express a range of L-Type Ca^2+^ channel splice variants. TSA significantly induced expression of the exon-8 variant (Left Panel; ^*^p < 0.05) whilst also reducing expression of a number of other splice variants (Right Panel; ^*^p < 0.05). TNF had no effect on splice variant expression. **(C)** Representative gel of the RT-PCR products. **(D)** Neither TNF nor TSA influenced the expression of GAPDH.

In our previous studies, we have reported differential effects of both TNF and TSA on signaling pathways in primary myometrial myocytes (Chapman et al., 2005; Webster et al., 2013). In the present study we sought to determine if such compounds could also influence expression of the MaxiK mRNA. Total cellular RNA was amplified using primers specific for a region within MaxiK that is conserved in all splice variants. As such, this would give an indication of overall expression of the mRNA for this channel and how TNF and TSA affected this. Figure 3A demonstrates that TSA was seen to induce a significant reduction in expression of MaxiK mRNA (Figure 3A, white bar). In contrast to this, TNF was seen to induce an increase in total MaxiK mRNA expression but this did not reach significance (Figure 4A, gray bar). Neither treatment influenced the expression of GAPDH (Figure 3D).

The MaxiK channel is documented to have a number of splice variants (Curley et al., 2004). Consequently, it was important to determine if the expression of such variants also occurred in primary human myometrial myocytes. Moreover, it was also salient to determine which of these variants were sensitive to TSA treatment. Total cellular RNA was amplified using primers specific for the different splice sites published for the MaxiK open reading frame. Primary myometrial myocytes were seen to express a number of different splice variants M1 (including both the MK44 variants which either express a 132 bp insert between exons 1 and 2 or have this insert omitted); M2 (+exon 11 and 12); (M3 (+exon 19); M4 (STREX-2); and M5 (+exon 29) (Figures 3B,C).

When cultures were subsequently exposed to either TNF or TSA, TSA significantly decreased the expression of the MK44 splice variant in comparison to un-stimulated cultures and the ratio between the MK44 variant containing the 132 bp insert and the variant lacking this insert was also significantly reduced with TSA stimulation. TNF had no effect on either the expression of the MK44 variant or on the ratio between the insert and insert-less forms of the variant (Figure 3B). TNF did, however, induce repression of the M3 (+ exon 19) variant. We also observed a similar TSA-induced repression of both the M3 (+ exon 19) variant and the M4 (STREX-2) variant (Figure 3B). The observed reduction in STREX-2 levels, while statistically significant, was, however, small in magnitude and the relevance of this remains unclear at present. Representative gels of each treatment are illustrated in Figure 3C. No change in GAPDH was observed with either treatment (Figure 3D).

### L-Type Ca^2+^ channel splice variants are expressed in non-laboring term pregnant myometrium and are down-regulated by trichostatin-A

Next, we determined if these compounds also influence expression of the LTCC mRNA. Total cellular RNA was amplified using primers specific for a region within LTCC that is conserved in all splice variants. As such, this would give an indication of overall expression of the mRNA for this channel and how TNF and TSA affected this. Figure 4A demonstrates that TSA was seen to induce a reduction in expression of LTCC mRNA but this did not reach significance (Figure 4A, white bar). In contrast to this, TNF was seen to induce a significant increase in total LTCC mRNA expression (Figure 4A, gray bar). Neither treatment influenced the expression of GAPDH (Figure 4D).

The LTCC is documented to have a number of splice variants (Tang et al., 2004). Consequently, it was important to determine if the expression of such variants also occurred in primary human myometrial myocytes. Moreover, it was also salient to determine which of these variants were sensitive to TNF treatment. Total cellular RNA was amplified using primers specific for the different splice sites published for the LTCC open reading frame. Primary myometrial myocytes were seen to express a number of different splice variants (Figures 4B,C). Briefly, the myocytes express the splice site 4 variant containing exon 8^*^ this variant has lower DHP sensitivity, more rapid activation and slower deactivation kinetics than the alternative exon 8-containing variant (Soldatov, 1992). Two splice site 10 variants were observed one containing exons 30, 31, 32, 33 and 34 and a second containing exons 30, 31, 33 and 34 these two variants affect the size and rigidity of the S3 to S4 linker segments. It has been suggested that shorter linker segments result in channels with slower gating kinetics while longer linking segments result in channels with faster gating kinetics (Perez-Reyes et al., 1990; Yang et al., 2000). Of the two variants found in the primary cells the one containing 30, 31, 33 and 34 is the shorter variant and was found in approximately 80% of the RNA. Finally the splice site 11 variant containing exons 40, 41, 42 and 43 was also observed.

When cultures were subsequently exposed to either TNF or TSA, TSA resulted in the novel expression of LTCC splice variants utilizing exon 8 (Figure 4B; Left Panel). Un-stimulated cells and those treated with TNF exclusively utilize exon 8^*^; after treatment with TSA, however, approximately 45% of the RNA expressed contained the exon 8 variant. The utilization of exon 8 leads to the expression of channels with higher DHP sensitivity and slower activation and more rapid deactivation curves (Figures 4B,C).

TSA also resulted in the down regulation of both forms of the splice site 10 variant while TNF had little effect (Figures 4B,C). The effect of TSA is what would be expected as the faster gating kinetics of the longer form would mean that it would take less time for the channels to fully open and hence would increase the influx of calcium and would promote contraction. The effect of a decreased expression of this variant would therefore lead to relaxation (Figures 4B,C). Neither treatment influenced the expression of GAPDH (Figure 4D).

TSA also resulted in a down regulation of the splice site 11 variant. All the treatment groups expressed the same variant at splice site 11, this variant contained exons 40, 41, 42 and 43 with no additions or deletions. After TSA treatment the expression of this variant was significantly reduced, there was, however, no alternative variant expressed (Figures 4B,C).

## Discussion

At the end of pregnancy the myometrium switches from a state of electrical quiescence seen throughout gestation to the contractile state characteristic of parturition which then culminates in the birth of the baby. Although the molecular basis of contraction is understood, the triggers which move the myometrium from the quiescence to the contractile state seen at parturition remain to be defined.

Myometrial electrical quiescence state is characterized by slow wave potentials where the membrane potential cycles between depolarizations and repolarizations without reaching the threshold level for action potential generation (Parkington et al., 1999). However, in myometrium obtained at term gestation, slow wave potentials become frequent and synchronized action potentials during which the membrane potential rapidly rises and falls, causing the muscle to contract (Wilde and Marshall, 1988; Khan et al., 1993b).

Both the quiescent state and the coordination of contractions are thought to be mediated through mechanisms that involve ion channels. Therefore we postulate that the balance between the MaxiK channel and the LTCC is of key significance in the switch from electrical quiescence to activation and synchrony of contractions.

### The MaxiK channel

This research has demonstrated that term myometrium contains a number of different MaxiK channel splice variants including MK44 and STREX-2. The MK44 variant comprises a 132 bp insertion, which has previously been described by Korovkina et al. (2001). Variants containing the insert have a diminished calcium and voltage sensitivity; the inserted sequence itself has been shown to contain both a phosphorylation and a myristylation site. This variant can also undergo proteolytic cleavage after which the N terminal is located on the membrane while the C-terminal is held with the endoplasmic reticulum. The C terminal is released after release of calcium from sarcoplasmic reticulum based stores and re-constitutes with the N-terminus on the membrane. The presence of this variant shows that there are MaxiK channels with diminished calcium and voltage sensitivity within the myometrium and is in agreement with electrophysiological results from our studies that demonstrate altered calcium and voltage-sensitivity of the MaxiK channel in laboring human myometrium (Khan et al., 1993b, 1997). Curley et al. (2004) demonstrated that the variant containing the 132 bp insert increased in laboring myometrium; this, however, was not evident in our study.

The MaxiK Splice site 4 consists of the differential utilization of exons 22 and 23. The variant present in the myocytes contained both exons 22 and 23 and so is the STREX-2 variant (Xie and McCobb, 1998). The STREX-2 variant has increased mechano-sensitivity and inhibition by hypoxia. Interestingly the STREX insert also contains a cAMP dependant Protein Kinase A (PKA) consensus motif which when phosphorylated facilitates membrane depolarization hence promoting the contractile phenotype of the cells.

### The L-Type Ca^2+^ channel

We have demonstrated that term myometrium expresses pro-contractile splice variants of the LTCC, including Splice site 4 containing exon 8^*^, Splice site 10 containing exons 30,31,32 and 34 and splice site 11 containing exons 40, 41, 42 and 43. LTCC Splice site 4 is comprised of the mutually exclusive use of exon 8 or 8^*^. The PCR results show that unstimulated term myocytes express exon 8^*^. The expression of 8^*^ results in a channel with decreased DHP sensitivity, rapid activation and slow deactivation kinetics (Goodwin et al., 1999). The kinetics of this channel variant would promote contraction.

The LTCC Splice site 10 is a complex site which involves the differential usage of exons 30, 31, 32, 33, and 34 which result in different length of linking loop between IV S2 and S4. The uterine myocytes express two forms of this variant- a longer one containing exons 30, 31, 32, 33, and 34 and a shorter one containing exons 30, 31, 33, and 34. The longer form containing exon 32 correlates well with SVI3B, which has been shown to have increased excitation-contraction coupling (Huang et al., 2006) and so the less abundant expression of this form would decrease the excitability of the cell and promote relaxation.

The LTCC Splice site 11 encompasses exons 40, 41, 42, and 43, variants in this region include use of exon 40A (exon40 − 19 bp), +125 bp (exon 40B), exon 41A and exon 43 +132 bp or the use of exons 40, 41, 42, and 43 (Soldatov, 1994). The additional 19 bp found in exon 40 in comparison to exon 40A are thought to modulate the tethering of calmodulin to the C-terminal and impact on the calcium dependant inactivation of the channel (Gerhardstein et al., 2000). The splice site 11 variant found to be expressed in the myocytes contained exons 40, 41, 42, and 43. The variant found at splice site 11 is the same variant Kepplinger et al. reported and named α1C_77_ (Kepplinger et al., 2000). Calcium-dependant inactivation was found to be the highest in the α1C_77_ variant (Soldatov et al., 1997). Also, this variant was found to be more efficiently targeted to the cell membrane and had higher conductance and open probability than other variants at this site (Kepplinger et al., 2000). The expression of this variant would push the cell toward a more contractile state.

Following TSA stimulation there was an overall reduction in the expression of variants at splice sites L10 and L11/12, there are two explanations for this, firstly the reduction in total LTCC RNA as a result of TSA treatment may result in an apparent reduction in this particular splice variant. We have compensated for this possibility by expressing the level of splice variant expression as a percentage of the total channel expression within the same treatment group. The second possibility is that the RNA is truncated (Snutch et al., 1991). There are two splice variants which have not been looked at in this study, of specific interest here is the possibility of a 4nt insertion into the 5′ end of exon 3. This insertion shifts the reading frame giving rise to a premature stop codon and resulting in the production of only the N terminus of the LTCC (Snutch et al., 1991; Soldatov, 1992). If this is the reason for the reduction in the splice site L10 and L11/12 variants then it would appear that TSA causes an increase in truncated protein.

The possible presence of a truncated LTCC comprising only the N-terminal domain is interesting as the C-terminus has been shown to exert an inhibitory role over the channel (Hell et al., 1996) and so a functional channel lacking the C-terminus will open more readily and support contraction. It is also possible that the putative truncated channel is silent. Either way this is certainly worthy of further investigation in understanding the control of myometrial contractility. These findings provide experimental evidence that toward the end of gestation, myometrium is primed ready to contract based on the channel variants present. Further work, however, directly comparing pregnant, non-laboring human myometrium to actively laboring tissue would be required to confirm this thesis.

### The action of TNF

The initiation of the contractile state seen at parturition is characterized by an influx of pro-inflammatory cytokines such as IL-1β, IL-6, and TNF (Romero et al., 1992; Opsjln et al., 1993; Keelan et al., 1999; Osman et al., 2003). This influx leads to the activation of NFκB which in turn promotes the expression of pro-contractile genes (Mendelson, 2009) as well as repressing those genes promoting myometrial quiescence such as Gαs (Chapman et al., 2005; Webster et al., 2013). As such, our observations from the mono-layer contraction studies demonstrating the contractile action of TNF and the pro-relaxant function of TSA are in broad agreement with the published data on the effects of TNF and TSA on contractility of isolated smooth muscle strips (Sadowsky et al., 2006; Fitzgibbon et al., 2009; Europe-Finner et al., 2013; Webster et al., 2013).

Whilst studying the function of the individual TNF receptors, TNFR1 and TNFR2, was outside the scope of this study, it is important to consider which receptor the TNF signal utilizes. For example, potassium channels can directly interact with TNFR1, and in airway smooth muscle it was shown that a change in contractile response induced by TNF was via TNFR1 signaling (Amrani et al., 2000; Tliba et al., 2004; Bronstein-Sitton, 2006; Wang et al., 2011). This study within the myometrium suggests that TNF signaling is through TNFR1 (Leroy et al., 2007). Finally, a study in cardiac myocytes demonstrates complex cross talk between TNFR1 and TNFR2 to control calcium transients (Defer et al., 2007).

TNF is a potent activator of NFκB (Cookson and Chapman, 2010); interestingly, the promoter of the MaxiK channel α subunit contains NFκB motifs which are enriched with the RelA-containing NFκB dimers after 1 h exposure to TNF (Cookson, 2010). Together, these data suggest that the myometrium may respond to such pro-inflammatory agents and permit transient changes in the pattern of channel expression associated with cellular exposure to cytokines (Dhulipala and Kotlikoff, 1999; Lu et al., 1999).

In terms of calcium flux, using hippocampal neurons, TNF stimulation has been shown to increase calcium current density by around 20% and this increase can be attributed to an increase in LTCC current (Furukawa and Mattson, 1998). Furukawa et al. went on to show that this increase was dependant on NFκB activation as inhibition of NFκB resulted in repression of these calcium current increases.

Calcium sensitization has also been suggested as playing a role in the switch from quiescence to contractility. Calcium sensitization is the phenomenon by which a given concentration of intracellular calcium results in a larger than expected force of contraction (Ratz et al., 2005; Arthur et al., 2007; Wray, 2007). TNF has been shown to enhance Ca^2+^ responsiveness tenfold in airway smooth muscle. It has been shown that TNF may cause this increased Ca^2+^ sensitivity through inducing increased IP_3_ turnover and hence increased release of Ca^2+^ from intracellular stores.

In this study TNF was shown to significantly increase the transcription of the MaxiK channel. This would initially seem at odds with TNF promoting contraction, however, an up-regulation of transcription does not always correspond to an up-regulation of protein levels. Kim et al. demonstrated this lag between MaxiK RNA and protein up-regulation in chick cochlear development and determined that it was due to delays in protein synthesis and trafficking/scaffolding of the channel subunits (Kim et al., 2010). A similar delay may be occurring in the myometrium.

The expression of the MaxiK STREX-2 variant was unchanged with TNFα stimulation, however, Gαs is known to decrease during labor (Europe-Finner et al., 1994) and as a result of this the level of PKA would decrease. This decrease in PKA would result in decreased phosphorylation of the MaxiK channel, but any phosphorylation in the presence of STREX-2 would facilitate membrane depolarization and support contraction (Tian et al., 2004). This support of contraction would decrease as the levels of PKA decrease. Hypothetically this could lead to support for contraction while the PKA levels are high at the start of parturition, then a gradual switch to support quiescence as the PKA levels drop leading to the termination of parturition.

TNF also resulted in a significant up regulation of the LTCCs, while the overall splice variant profile was unchanged.

### The action of TSA

Trichostatin A is a potent histone deacetylase inhibitor (HDACi) which results in an increase in histone acetylation and can alter gene expression by preventing DNA transcription factors from accessing the DNA. It can also induce global protein acetylation (Spange et al., 2009). Studies have shown that TSA also has a potent inhibitory effect of myometrial contractility strips (Lu et al., 1999; Moynihan et al., 2008; Europe-Finner et al., 2013; Webster et al., 2013), although the relaxant effect induced by TSA could be overcome by pro-inflammatory agents such as TNF (Webster et al., 2013). While TSA is a functional HDACi it can, of course, modulate non-histone proteins acetylation too and this has been reviewed in the myometrium and broader research fields (Spange et al., 2009; Europe-Finner et al., 2013). Indeed, the non-nuclear lysine deacetylase (KDAC) KDAC8 modified Hsp20 acetylation profile in such a manner that myometrial contractility was subsequently inhibited (Karolczak-Bayatti et al., 2011). Consequently, we speculate that in myometrial cells, such putative global acetylation, coupled with chromatin-based effects, could influence the balance of channel splice variant formation.

The transition of the myometrium from the quiescent state to the contractile state seen at parturition has been shown to be facilitated by the down regulation of the hCG/LH receptors. Moynihan et al. (2008) demonstrated that TSA promoted the transcriptional activation of the hCG/LH receptor gene and hence the maintenance of quiescence (Phillips et al., 2005; Moynihan et al., 2008). It has also been shown that the maintenance of myometrial quiescence is facilitated by the expression of Gαs. Acetylation of the Gαs promoter by Cyclic-AMP Response Element Binding Protein (**C**REB) Binding Protein (CBP) is necessary for its expression within the myometrium and therefore TSA may also act by preventing the deacetylation of the Gαs promoter and hence promote the expression of Gαs and the maintenance of myometrial quiescence (Webster et al., 2013).

This research suggests a further possible mechanism for the pro-quiescent action of TSA via its effect on the expression of both the MaxiK and LTCCs. TSA resulted in the significant down regulation of both channels. After TSA stimulation the LTCC expressed variants containing exon 8 and those containing 8^*^, the presence of variants containing exon 8 would result in a less contractile phenotype. This reduction in MaxiK and LTCC transcription would result in cells much less responsive to calcium and so they would be much less contractile.

## Conclusion

This research suggests that increasing TNF levels at parturition promote the increased calcium sensitivity of the myometrium. We suggest this occurs through expression of MaxiK channel variants which have both decreased calcium and voltage sensitivity, in conjunction with LTCCs displaying rapid activation, slow deactivation kinetics and increased excitation-contraction coupling within the myocytes (Soldatov et al., 1997; Goodwin et al., 1999; Korovkina et al., 2001; Tian et al., 2004; Huang et al., 2006).

The combined effect of TNF on IP3 turnover and subsequent release of Ca^2+^ from intracellular stores, the increase of transcription of the LTCCs and the expression of MaxiK channel variants with decreased calcium and voltage sensitivity may serve to tip the fine balance of the channels allowing action potentials to be generated and propagated across the tissue resulting in contraction.

TSA was seen to promote a relaxatory effect on the cells, this is appears to be through the significant reduction in transcription of both channels resulting in cells that are less excitatory. TSA has been suggested as a potential tocolytic due to its relaxatory effect on the myometrium. More work, however, is required to determine the combined effect of TSA and TNF on myometrial excitability and how these functions involve protein acetylation, within chromatin and globally within the cyctosol, before such HDAC and KDAC inhibitors could be used as successful clinical interventions for pre-term labor.

## Funding

This work was funded by The Jessop Wing Small Grants Scheme (Grant Nos. OGN/13/01 to Sarah L. Waite and OGN/11/05 and OGN/12/02 to Neil R. Chapman).

### Conflict of interest statement

The authors declare that the research was conducted in the absence of any commercial or financial relationships that could be construed as a potential conflict of interest.
